# Asthma families show transmission disequilibrium of gene variants in the vitamin D metabolism and signalling pathway

**DOI:** 10.1186/1465-9921-7-60

**Published:** 2006-04-06

**Authors:** Matthias Wjst, Janine Altmüller, Theresia Faus-Kessler, Christine Braig, Margret Bahnweg, Elisabeth André

**Affiliations:** 1 Institut für Epidemiologie, GSF – Forschungszentrum für Umwelt und Gesundheit, Ingolstädter Landstrasse 1, Neuherberg/Munich, Germany; 2 Institut für Experimentelle Genetik GSF – Forschungszentrum für Umwelt und Gesundheit, Ingolstädter Landstrasse 1, Neuherberg/Munich, Germany

## Abstract

The vitamin D prophylaxis of rickets in pregnant women and newborns may play a role in early allergic sensitization. We now asked if an already diseased population may have inherited genetic variants in the vitamin D turnover or signalling pathway.

Serum levels of calcidiol (25-OH-D_3_) and calcitriol (1,25-(OH)_2_-D_3_) were retrospectively assessed in 872 partipants of the German Asthma Family Study. 96 DNA single base variants in 13 different genes were genotyped with MALDI-TOF and a bead array system. At least one positive SNP with a TDT of p < 0.05 for asthma or total IgE and calcidiol or calcitriol was seen in IL10, GC, IL12B, CYP2R1, IL4R, and CYP24A1. Consistent strong genotypic association could not be observed. Haplotype association were found only for CYP24A1, the main calcidiol degrading enzyme, where a frequent 5-point-haplotype was associated with asthma (p = 0,00063), total IgE (p = 0,0014), calcidiol (p = 0,0043) and calcitriol (p = 0,0046).

Genetic analysis of biological pathways seem to be a promising approach where this may be a first entry point into effects of a polygenic inherited vitamin D sensitivity that may affect also other metabolic, immunological and cancerous diseases.

## Background

Asthma is a chronic inflammatory condition of the airways, variable airway obstruction and elevated serum IgE levels of unclear pathogenesis [1]. A hypothesis relating early vitamin D supplementation and induction of later allergy has initially been postulated as the main cholecalciferol metabolite calcitriol may suppresses dendritic cell maturation and consecutive development of Th1 cells [2] which is now supported by in vitro, animal and human studies [3,4].

**Figure 1:**
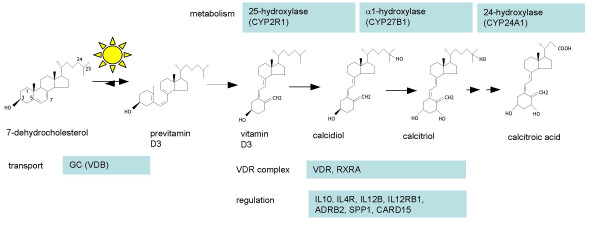
Pathway diagram of genes tested for association.

Exposure studies in humans, however, are difficult as nearly all newborns in Western countries are now being exposed in utero or during the first year of life to vitamin supplements [5,6]. We now asked if there are DNA sequence variants that are associated with higher or lower levels of vitamin D metabolites. As it is unlikely that any complex disease is determined by variants in a single gene we tested the main genes that code for enzymes in the metabolic pathway of vitamin D conversion (Figure 1).

## Methods

### Study population

The German Asthma Family Study collected affected sib pairs in 26 paediatric centres in Germany and Sweden for a two-stage genome-wide linkage scan [7,8]. In these families at least two children were required with confirmed clinical asthma, while prematurity or low birth weight of the children were excluded, along with any other severe pulmonary disease. All affected children over age 3 had a history of at least 3 years of recurrent wheezing and with no other airway disease diagnosed. Unaffected siblings were also sampled if they were at least 6 years old and eligible for pulmonary function testing. Each study participant signed a consent form. All study methods were approved by the ethics commission of "Ärztekammer Nordrhein-Westfalen".

A complete pedigree of the family was drawn and information collected in a questionnaire. Participants were examined for several closely associated phenotypes. Pulmonary function tests were performed by forced flow volume tests and bronchial challenge was done by methacholine (discontinued in the second stage of the study) as reported earlier [7,8].

**Figure 2:**
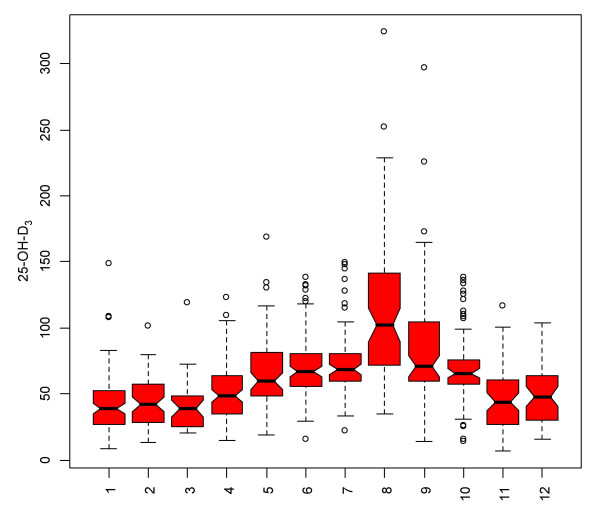
Median, quartile and outlier of 25-OH-D_3 _serum levels in 872 participants of the German Asthma Family Study with white skin colour by month of examination.

The current analysis differs from previous publications [7,8]. We excluded here all families with at least one member of non white skin colour (families 2, 14, 16, 19 to 21 and 27 to 32) as these individuals had considerable lower levels of 25-OH-D_3 _(data not shown) compared to all other participants (Figure 2).

Total IgE was determined with an ELISA (Pharmacia Diagnostics, Uppsala, Sweden). 25-OH-D_3 _was determined with an enzymatic immunoassay (OCTEIA 25-Hydroxy Vitamin D kit, Immunodiagnostic Systems IDS, Frankfurt, Germany) that has a working range of 6–360 nmol/L, an intra-assay of 8% and inter-assay variation of 10% with a 100% specificity for 25-OH-D_3 _and 75% specificity for 25-OH-D_2 _according to the manufacturer. 1,25-OH2-D_3 _was determined by immunoextraction followed by an enzyme-immunoassay (OCTEIA 1,25-Hydroxy Vitamin D kit, Immunodiagnostic Systems IDS, Frankfurt, Germany) that has a working range of 6–500 pmol/L, a 100% specificity for 1,25-OH_2_-D_3 _and 0,009% specificity for 25-OH-D_2_. 25-OH-D_3 _values reported are the mean of a duplicate analysis while due to limited serum availability only single assays have been performed for 1,25-OH_2_-D_3_.

### Control population

191 anonymized DNAs were selected randomly from the ECRHS II study [9] to fill in remaining slots on the genotyping plates. These DNA samples served as population-based controls to test if the parents of the famillies had different allele spectrum.

### DNA preparation and genotyping

DNA was isolated from peripheral white blood cells using Qiamp (Qiagen, Germany) or Puregene isolation kits (Gentra Systems, Minneapolis, MN, USA).

Genes were selected as coding either for key enzymes in the vitamin D conversion pathway or being regulated by vitamin D metabolites [10]. SNPs were being picked more or less randomly either for tagging haplotypes or being functional relevant [11]. Most SNPs were genotyped using MALDI-TOF mass spectrometry of allele-specific primer extension products generated from amplified DNA sequences (MassARRAY, SEQUENOM Inc., San Diego, CA, USA). A few SNPs were also genotyped at Illumina (San Diego CA, USA) by use of the Sentrix bead arrays. VDR [12] and IL4R [13] SNP results have been published earlier and are reanalysed here for the vitamin D levels. The following SNPs were analyzed (genotyping details upon request): rs3024498 (IL10), rs3024493 (IL10), rs1518111 (IL10), rs10000076 (IL10), rs1800872 (IL10), Il10-571CA (IL10), rs1800895 (IL10), rs1800894 (IL10), rs1800896 (IL10), rs1800893 (IL10), rs705120 (GC), rs222040 (GC), rs7041 (GC), rs4752 (GC), rs222011 (GC), rs221999 (GC), rs6811536 (SPP1), rs4754 (SPP1), rs1042714 (ADRB2), rs1800888 (ADRB2), rs1368439 (IL12B), rs3212227 (IL12B), rs2853697 (IL12B), rs3213119 (IL12B), rs2853696 (IL12B), rs2853694 (IL12B), rs2288831 (IL12B), rs3213096 (IL12B), rs2569254 (IL12B), rs3181216 (IL12B), rs3212220 (IL12B), rs3212218 (IL12B), rs1433048 (IL12B), rs2546890 (IL12B), rs3132299 (RXRA), rs877954 (RXRA), rs1045570 (RXRA), rs10500804 (CYP2R1), rs1562902 (CYP2R1), rs10766197 (CYP2R1), rs2853563 (VDR), rs731236 (VDR), rs7975232 (VDR), rs1544410 (VDR), rs2239185 (VDR), rs987849 (VDR), rs1540339 (VDR), rs3819545 (VDR), rs3782905 (VDR), rs2239186 (VDR), rs2228570 (VDR), rs1989969 (VDR), rs2853564 (VDR), hCV2880804 (VDR), rs238532 (CYP27B1), rs2072052 (CYP27B1), rs1048691 (CYP27B1), rs4646537 (CYP27B1), rs4646536 (CYP27B1), rs8176345 (CYP24A1), rs703842 (CYP27B1), I50V (IL4R), rs2234897 (IL4R), rs1805011 (IL4R), C406R (IL4R), rs1805015 (IL4R), Q551R (IL4R), rs1805016 (IL4R), rs10000306 (CARD15), rs2076753 (CARD15), rs2066842 (CARD15), rs2066843 (CARD15), rs2076756 (CARD15), rs10000331 (CARD15), rs3135499 (CARD15), rs3135500 (CARD15), rs375947 (IL12RB1), rs447009 (IL12RB1), rs436857 (IL12RB1), rs2045387 (IL12RB1), rs8118441 (CYP24A1), rs751089 (CYP24A1), rs6068816 (CYP24A1), rs4809958 (CYP24A1), rs2244719 (CYP24A1), rs2296241 (CYP24A1), rs17219266 (CYP24A1), rs6022999 (CYP24A1), rs17219315 (CYP24A1), rs11699278 (CYP24A1), rs2762942 (CYP24A1), rs2248137 (CYP24A1), rs2762943 (CYP24A1), rs2585427 (CYP24A1), rs2248359 (CYP24A1) and rs2426496 (CYP24A1).

### Data handling and statistical analysis

Clinical data and genotypes were all transferred to a SQL 2000 database and checked for completeness, paternity, and Hardy-Weinberg equilibrium. Further analyses were performed using R 2.0 statistical software[14]. Linkage disequilibrium was determined by Haploview [15] using the Gabriel method for block definition. TDT association for quantitative and qualitative traits was done with SIBPAIR [16] using the TDT option for qualitative and the Haseman-Elston regression for quantitative traits. Family-based haplotype association analysis was performed by FBAT [17] using a dominant model.

### Bioinformatics

SNP information was obtained from dbSNP [18], Innate Immunity PGA [19] and UCSC genome browser [20]. SNP selection was done with the help of Perlegen [21] and Hapmap [22] data. Sequence context annotation was done by SNPper [23], PUPA [24], TAMAL [25] and SNPPi [26]).

## Results

The total sample consisted of 947 individuals from 224 families where 872 serum measurements of 25-OH-D_3_, 876 1,25- OH_2_-D_3 _and 934 total IgE measurements could be performed. After exclusion of non-white families 903 individuals from 201 families remained under analysis with 812 assays of 25-OH-D_3_, 807 1,25- OH_2_-D_3 _and 903 total IgE.

Clinical details of the families are given in table 1. Mean 25-OH-D3 level in children was 68 nmol/l (s.d. 38 nmol/l). 50% of values fell below and 17% above the Merck manual reference range of 62.4 to 99.8 nmol/l. Mean 1,25- OH_2_-D_3 _in children was 102 pmol/l (s.d. 38 nmol/l). 3% of values fell below and 40% above the Merck manual reference range of 48.4 to 108 pmol/l. The highest measured value was 257 pmol/l in two children from unrelated families.

**Table 1:** Clinical characteristics of the included 210 families of the German Asthma Family

	Parents	Children
	n/N or mean/s.d.	n/N or mean/s.d.
age/years)	43.2/6.1	13.6/4.6
female sex	204/408 (50.0%)	207/474 (43.7%)
height(cm)	172.3/9.6	149.0/19.6
weight(kg)	76.4/15.1	42.8/17.2
		
asthma diagnosis	99/408(24.3%)	416/474 (87.8%)
Eczema	51/382(13.4%)	184/448 (41.1%)
allergic rhinitis	163/406 (40.1%)	280/474 (59.1%)
		
D.pter. (D1) > 0.5 kU/l	109/340 (32.1%)	243/422 (57.6%)
D.far. (D2) > 0.5 kU/l	103/341 (30.2%)	236/422 (55.9%)
grass(GX1) > 0.5 kU/l	129/340 (37.9%)	293/422 (69.4%)
birch (T3) > 0.5 kU/l	122/339 (36.0%)	213/423 (50.4%)
mean positive RASTs	2.6/2.9	4.8/3.5
Eosinophils 10^3^/μl	0.2/0.2	0.4/0.4
IGE kU/l	185.0/371.8	480.6/701.3
mean RAST > 0.5 kU/l	2.6/2.9	4.8/3.5

**Figure 3:**
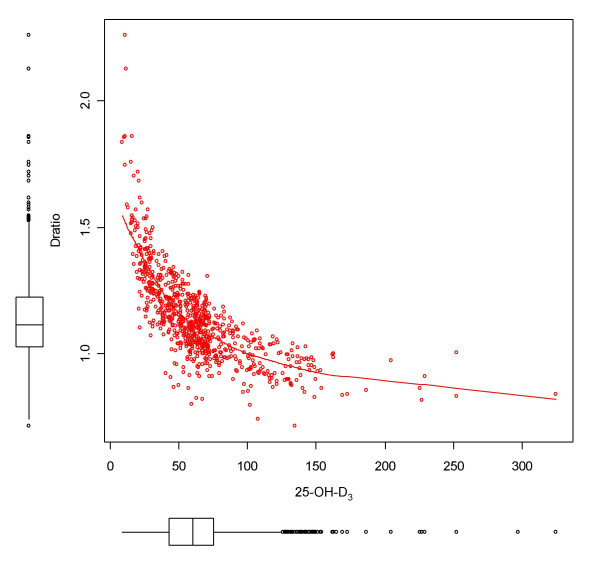
D-ratio (log(1,25-OH_2_-D_3_) [pmol/l]/log(25-OH-D3) [nmol/l]) versus 25-OH-D_3 _in in 867 participants of the German Asthma Family Study with white skin colour.

There were no major differences in serum levels between children and parents. There was also no major influence by sex or age. An important factor, however, was found with month of examination representing seasonal sun exposure in mid Europe (Figure 2). Even after serum storage of 10 years, the individual 25-OH-D_3 _levels followed a clear time course with a major peak in August. The hormonal form 1,25-OH_2_-D_3 _did not vary over the course of the year, as the conversion rate decreased with higher levels of 25-OH-D_3 _(Figure 3).

The overall heritability index H^2 ^for 25-OH-D_3 _was 80.3% while the H^2 ^for 1,25-OH_2_-D_3 _was only 30.0% [27]. There was neither an association of 25-OH-D3 and total IgE nor an association of 1,25-OH_2_-D_3 _and total IgE levels.

13 genes were selected for genotyping (IL10, GC, SPP, ADRB2, IL12B, RXRA, CYP2R1, VDR, CYP27B1, IL4R, CARD15, IL12RB1, CYP24A1) and could be successfully completed for 96 SNPs. 4 of these SNPs were not in Hardy-Weinberg equilibrium: rs221999 (GC, P = 0,0299), rs10500804 (CYP2R1, P = 0,0498), rs10766197 (CYP2R1, P = 0,0100) and rs2248359 (CYP24A1, P = 0,0299). SNP rs221999 was also not in Hardy-Weinberg equilibrium in controls. The population-based controls showed similar allele frequencies compared to the family samples except for SNPs rs4754, rs2288831 and rs3819545.

SNP allele transmission in 7 of the tested 13 genes showed a p-value of less than 0.05 when testing for 25-OH-D_3 _levels (IL10, GC, ADRB2, CYP2R1, IL4R, IL12RB1 and CYP24A1, see table 2) while only 3 showed transmission disequilibrium with 1,25-OH2-D3 (IL10, IL12B and CYP24A1). SNPs in 5 genes showed a p-value < 0.05 with asthma (IL10, IL12B, VDR, CARD15 and CYP24A1). Most significances, however, were weak. For 96 SNPs we would expect 4.8 tests to be positive for each trait which was exceeded by testing asthma (N = 8), total IgE (N = 13), 25-OH-D_3 _(N = 8) but not 1,25-OH_2_-D_3_(N = 3). Only 2 SNP showed an effect with both traits, one in CYP2R1 (rs10766197) and one in IL4R (rs1805011). rs10766197 is situated in the CYP2R1 promotor; while rs1805011 is leading to an Ala- > Glu amino acid exchange in the IL4 receptor.

**Table 2:** Transmission approach: TDT results of 30 from 96 tested SNPs in 210 families of the German Asthma Family Study. Shown are only SNPs with at least one TDT results of p < 0.05. The underlined 11 SNPs also appear in table 3.

SNP	Gene	Genomic position HG17	MAF	P asthma	P log(IgE)	P log(25- OH-D3)	P log(1,25- OH2-D3)
rs3024498	IL10	1203329924	0,26	0,2654	0,4625	**0,0093**	0,4479
rs1518111	IL10	1203333040	0,22	0,7087	**0,0487**	0,4962	0,6031
rs10000076	IL10	1203334410	0,02	0,3711	0,2364	0,2374	**0,0030**
Il10-571CA	IL10	1203334805	0,24	**0,0247**	0,3310	0,5889	0,3857
rs1800895	IL10	1203334867	0,01	**0,0039**	0,5237	0,1152	0,3891
rs1800894	IL10	1203335061	0,03	0,7773	0,5154	**0,0101**	0,1727
rs222040	GC	4072981967	0,43	0,9211	**0,0163**	0,3829	0,0662
rs7041	GC	4072983369	0,43	0,8812	**0,0249**	0,3884	0,0827
rs221999	GC	4073014083	0,24	0,1475	0,2755	**0,0027**	0,1920
rs1042714	ADRB2	5148186666	0,40	0,8532	0,8492	**0,0139**	0,7524
rs1433048	IL12B	5158688423	0,18	0,3452	0,5814	0,4184	**0,0339**
rs2546890	IL12B	5158692478	0,45	**0,0236**	0,9066	0,5667	0,1599
rs10500804	CYP2R1	11014866849	0,45	0,2242	**0,0418**	0,0833	0,8352
rs1562902	CYP2R1	11014874792	0,43	0,1342	**0,0443**	0,1063	0,8049
rs10766197	CYP2R1	11014878456	0,48	0,4700	**0,0284**	**0,0459**	0,7258
rs7975232	VDR	12046525104	0,49	0,2484	**0,0400**	0,9235	0,9351
rs2239186	VDR	12046555677	0,19	**0,0350**	0,7521	0,9212	0,5459
rs1805011	IL4R	16027281373	0,11	0,2334	**0,0090**	0,1110	0,2684
C406R	IL4R	16027281467	0,15	1,0000	**0,0255**	0,6542	0,1249
rs1805015	IL4R	16027281681	0,16	0,8744	**0,0003**	0,4956	0,1197
Q551R	IL4R	16027281901	0,25	0,2482	**0,0017**	0,9661	0,8287
rs1805016	IL4R	16027282428	0,05	0,2076	**0,0082**	**0,0002**	0,7390
rs10000306	CARD15	16049288266	0,04	0,6473	**0,0417**	0,4739	0,5429
rs3135499	CARD15	16049323628	0,37	**0,0374**	0,2742	0,3929	0,8405
rs2045387	IL12RB1	19018061586	0,24	0,6826	0,7907	**0,0145**	0,3191
rs2296241	CYP24A1	20052219626	0,50	**0,0046**	0,9291	0,8917	0,5098
rs17219315	CYP24A1	20052221853	0,02	0,8658	0,6289	**0,0095**	0,3056
rs2762942	CYP24A1	20052222332	0,04	0,7140	0,1506	0,2030	**0,0397**
rs2248137	CYP24A1	20052223150	0,39	**0,0256**	0,8250	0,2567	0,9096
rs2248359	CYP24A1	20052224925	0,36	**0,0158**	0,9302	0,2284	0,8510

In a next step we performed multivariate regression in the parental dataset while adjusting for age, sex, and month of examination (table 3). This confirmed 11 SNPs already found in the family based-aproach; again, association results were weak. Some CARD15 variants had an asthma protective effect while IL12B SNPs carried risk alleles.

**Table 3:** Case-control approach: Multivariate regression results of 31 from 96 tested SNPs in 408 parents of the German Asthma Family Study adjusted for age, sex and month of examination. Shown are only SNPs with at least one p < 0.05 for heterocygotes and homocygote carriers of the minor allele. The underlined 11 SNPs also appear in table 2.

SNP	Gene	P asthma				P log(IgE)				P log(25-OH- D3)				P log(1,25-OH2-D3)			
		heterocygote		homocygote		heterocygote		homocygote		heterocygote		homocygote		heterocygote		homocygote	
		ß coeff.	P	ß coeff.	P	ß coeff.	P	ß coeff.	P	ß coeff.	P	ß coeff.	P	ß coeff.	P	ß coeff.	P
rs3024498	IL10	0,132	0,837	-0,140	0,822	0,875	**0,034**	1,026	**0,011**	0,002	0,984	0,068	0,575	-0,022	0,819	0,014	0,883
Il10571CA	IL10	1,731	**0,024**	1,225	0,080	0,446	0,362	0,241	0,608	0,171	0,290	0,252	0,110	-0,080	0,484	-0,090	0,421
rs6811536	SPP1	0,215	0,632	0,266	0,555	0,532	0,069	0,687	**0,019**	0,159	0,112	0,149	0,136	-0,048	0,528	-0,038	0,623
rs3213119	IL12B	-13,300	0,988	-13,765	0,988	2,221	0,149	2,950	**0,050**	-0,125	0,799	-0,124	0,795	-0,063	0,869	-0,090	0,808
rs2853694	IL12B	-0,295	0,394	-0,845	**0,020**	0,212	0,288	0,272	0,216	-0,076	0,241	-0,092	0,200	0,069	0,173	0,003	0,952
rs3181216	IL12B	0,729	0,058	0,972	**0,013**	-0,306	0,231	-0,300	0,238	0,086	0,322	0,191	**0,029**	0,090	0,180	0,051	0,446
rs1433048	IL12B	0,632	0,412	0,595	0,427	0,916	0,077	1,074	**0,034**	0,082	0,658	0,019	0,916	0,063	0,656	0,037	0,792
rs2546890	IL12B	-0,367	0,293	-0,808	**0,026**	0,166	0,391	0,272	0,199	-0,059	0,355	-0,074	0,291	0,002	0,969	-0,033	0,547
rs3132299	RXRA	-15,303	0,986	-15,118	0,987	0,803	0,181	0,383	0,517	0,032	0,873	0,108	0,587	0,319	**0,042**	0,366	**0,018**
rs877954	RXRA	-0,433	0,301	-0,258	0,551	0,345	0,163	-0,119	0,640	0,175	**0,029**	0,156	0,059	0,031	0,616	-0,003	0,965
rs10500804	CYP2R1	0,279	0,394	0,287	0,434	0,212	0,323	0,421	0,077	0,069	0,323	0,191	**0,014**	0,032	0,554	-0,001	0,982
rs1562902	CYP2R1	-0,071	0,846	0,020	0,960	-0,020	0,930	0,026	0,917	-0,108	0,143	-0,202	**0,011**	0,038	0,517	0,050	0,426
rs10766197	CYP2R1	-0,112	0,733	0,014	0,970	0,259	0,202	0,377	0,104	0,074	0,266	0,181	**0,017**	0,032	0,532	-0,018	0,757
rs1544410	VDR	-0,105	0,786	-0,268	0,496	-0,350	0,145	-0,149	0,544	0,041	0,592	0,111	0,165	-0,120	**0,040**	0,047	0,441
rs2239185	VDR	0,620	**0,041**	0,554	0,110	-0,184	0,352	-0,187	0,408	-0,022	0,730	-0,007	0,920	-0,110	**0,028**	-0,034	0,544
rs2239186	VDR	-0,274	0,669	0,289	0,641	0,638	0,121	0,558	0,160	-0,268	**0,040**	-0,163	0,193	-0,218	**0,036**	-0,156	0,119
rs2228570	VDR	0,708	0,104	0,834	0,058	0,481	0,108	0,653	**0,029**	-0,100	0,279	-0,073	0,435	-0,042	0,537	-0,001	0,988
rs238532	CYP27B1	-0,449	0,577	0,626	0,214	0,647	0,144	-0,075	0,813	-0,025	0,857	0,081	0,463	0,281	**0,009**	-0,063	0,456
rs703842	CYP27B1	-0,467	0,316	-0,391	0,401	0,549	**0,036**	0,525	**0,045**	-0,041	0,631	0,046	0,589	-0,064	0,335	-0,022	0,738
rs1805011	IL4R	-13,530	0,986	-13,774	0,986	-2,094	**0,024**	-1,752	0,053	0,559	**0,049**	0,497	0,073	0,193	0,382	0,212	0,329
rs2076753	CARD15	-1,152	**0,044**	-1,300	**0,023**	0,093	0,738	0,137	0,625	0,062	0,483	0,057	0,526	0,000	0,998	-0,059	0,397
rs2066842	CARD15	-1,260	**0,028**	-1,416	**0,013**	-0,039	0,884	0,083	0,757	0,037	0,669	0,014	0,871	0,028	0,673	-0,038	0,560
rs2066843	CARD15	-1,221	**0,033**	-1,301	**0,023**	-0,066	0,813	0,055	0,842	0,007	0,939	0,010	0,912	0,016	0,819	-0,041	0,544
rs2076756	CARD15	-1,396	**0,031**	-1,427	**0,027**	-0,142	0,627	0,073	0,800	0,077	0,410	0,055	0,552	0,035	0,627	-0,029	0,680
rs375947	IL12RB1	1,012	**0,011**	0,580	0,125	0,117	0,653	0,087	0,733	0,133	0,116	0,063	0,443	0,140	**0,027**	-0,011	0,858
rs2244719	CYP24A1	0,257	0,407	-0,029	0,931	-0,045	0,817	0,184	0,389	0,139	**0,029**	0,025	0,723	0,165	**0,001**	0,114	**0,031**
rs2296241	CYP24A1	0,042	0,889	0,548	0,125	0,101	0,609	-0,018	0,934	0,160	**0,012**	0,030	0,669	-0,058	0,236	0,039	0,463
rs2248137	CYP24A1	-0,104	0,776	-0,253	0,503	0,320	0,145	0,117	0,609	0,038	0,599	-0,024	0,748	-0,120	**0,028**	-0,131	**0,021**
rs2762943	CYP24A1	15,934	0,986	15,861	0,986	-3,140	**0,041**	-3,193	**0,036**	-0,146	0,763	-0,195	0,684	-0,878	**0,018**	-0,784	**0,033**
rs2248359	CYP24A1	-0,109	0,793	-0,460	0,245	0,230	0,345	0,057	0,805	-0,023	0,766	-0,019	0,803	-0,137	**0,025**	-0,112	0,056
rs2426496	CYP24A1	0,415	0,403	-0,091	0,848	-0,045	0,886	-0,225	0,464	0,207	**0,038**	0,165	0,091	-0,060	0,436	-0,076	0,314

Haplotypes were constructed from all significantly associated SNPs (table2). No significant association was found in any of the 13 genes except for CYP24A1 where a 5-point frequent haplotype (rs2296241:rs17219315:rs2762942:rs2248137:rs2248359) spanning both LD blocks of CYP24A1 was associated with a diagnosis of asthma (p = 0.001), total IgE (p = 0.001), 25-OH_2_-D_3 _(p = 0.004) and 1,25-OH_2_-D_3 _serum level (p = 0.005, table 4).

**Table 4:** CYP24A1 haplotype transmission results in 213 families of the German Asthma Family Study. Haplotype was formed from all SNPs with p < 0.05 in the TDT (table 2).

		h1	h2	h3	h4	h5	h6	h7
SNP	rs2296241	G	A	A	G	G	A	A
	rs17219315	A	A	A	A	A	A	G
	rs2762942	A	A	A	G	A	A	A
	rs2248137	C	G	C	C	G	G	G
	rs2248359	C	T	C	C	T	C	T
frequency		0,40	0,31	0,15	0,04	0,03	0,03	0,02

asthma	Z value	3,42	-2,11	-1,43	0,10	NA	NA	NA
	P value	**0,001**	**0,035**	0,153	0,919	NA	NA	NA
log(IgE)	Z value	3,19	-1,97	-1,26	-0,46	0,71	NA	-0,79
	p value	**0,001**	**0,049**	0,208	0,645	0,478	NA	0,432
log(25-OH-D3)	Z value	2,85	-2,25	-1,03	0,08	1,59	NA	-0,22
	P value	**0,004**	**0,025**	0,305	0,935	0,112	NA	0,823
log(1,25-OH2-D3)	Z value	2,83	-1,94	-1,38	0,10	1,50	NA	-0,09
	p value	**0,005**	0,052	0,167	0,922	0,134	NA	0,932

## Discussion

We have shown that serum 25-OH-D_3 _(calcidiol) levels -although highly influenced by environmental sunlight exposure- is a heritable trait in asthma families. In contrast, a major genetic influence on 1,25-OH_2_-D_3 _(calcitriol) levels could not be found, a finding that requires replication in further family and population-based studies.

The reason for this discrepancy is not fully clear as the conversion of 25-OH-D_3 _to 1,25-OH_2_-D_3 _is closely regulated by a direct feedback loop. It is generally agreed, however, that 25-OH-D_3 _reflects best the current vitamin D status [28]. Unfortunately standardized reference values for this age group are not available but values for 25-OH-D_3 _in children seem to be in the upper normal range [29]. An explanation therefore could be that a delayed downstream metabolism is leading to an (unintended) afflux or -also possible- that an increased peripheral demand needs a larger reservoir.

We observed a number of positive associations with single nucleotide polymorphisms. Although the selection of candidate genes was rather subjective, it turned out that some of the tested candidate genes are associated with both allergy and vitamin D metabolites. Statistical significance, however, was weak, and varied even with different analysis strategies and software packages (unpublished own observation). There was also no fully consistent pattern when comparing the family transmission and the case-control approach which makes it unlikely that any of the tested SNPs is already an important functional variant. The new associations may instead indicate the effects of physically closely related variants in these genes (which is also supported by the haplotype results of CYP24A1).

The associated candidate genes are of particular interest. CYP24A1 is the major enzyme of the calcitriol degradation pathway that showed nearly 100-fold increase after vitamin D treatment of rats [30]. Previous studies also suggest that CYP24A1 null mice cannot clear calcitriol efficiently [31] which would support the above mentioned afflux hypothesis. An alternative splicing variant in CYP24A1 has been described recently [32] leading to a truncated and catalytically dysfunctional protein while it is unclear if any of our tested SNPs will have functional relationship to this protein variant. Dark skinned Asian Indians seem to have increased 24-hydroxylase activity compared to white skinned Caucasians [33] whereas both skin colour and metabolic capacity seem to be adapted to less sun light exposure in Caucasians.

The evidence that the human CYP2R1 is a key vitamin D 25-hydoxylase is rather new [34] where the identity of the hepatic 25-hydroxylase has remained unclear for several decades. At least six CYPs can catalyze this step where the most viable candidates are CYP27A1 and CYP2R1 [34] with the renal enzyme responsible for 1-α-hydroxylation being CYP27B1. A loss of function mutation in CYP2R1 has also been described [34] and deserves further testing.

Variants in CYP2R1, CYP27B1 and CYP24A1 or other genes in the metabolic pathway of vitamin D have not been tested so far with asthma or allergy but several of the VDR-controlled genes tested here already have been associated with asthma and allergy. These include IL12B [35-37], IL12RB [38,39], IL10 [40], VDR [41,42,12], GC [43], ADRB2 [44], CARD15 [45] and IL4R [46].

Of these, IL12B is a particular interesting cytokines. Macrophage engulfed microorganism are leading to IL12p70 production, a heterodimer of IL12p40 (IL12B) and IL12p35 (IL12A), which is a primary inducer of Th_1 _cell development and a critical factor in the development of allergy [47]. Also IL10 seems to be important where production in circulating T cells from atopic asthmatics is maximally stimulated [48]; allergen specific IL10 producing T regulatory cells can inhibit allergen specific effector cells and represent an important line of defense in the allergic reaction [49]. Functional variants in these genes leading to human disease are not known so far.

The many positive but weak associations represent a common dilemma in complex disease. In asthma more than 75 genes have now been claimed to be associated [50] but none of them has been shown to contribute to risk in all populations studied [51]. Obviously there are only small genetic effects and a large heterogeneity; sometimes there is unidentified population stratification and there might be phenotyping and genotyping errors. Most likely, however, not the "center" SNPs have been choosen [11]. The current pathway based approach seems to be an alternative in particular when an environmental trait can be included. It is likely that some of the genes identified here are acting in concert to determine the overall vitamin D sensitivity.

Besides increasing sample size and testing additional populations, further work may concentrate on monitoring vitamin D supplementation by immunological readouts and the identification of contributing functional genetic elements. The present rediscovery of a genetic vitamin D sensitivity [52] may be an important step in allergy induction and also surmount many other diseases including type 1 diabetes, osteoporosis, tuberculosis, rheumatoid arthritis, multiple sclerosis, inflammatory bowel diseases, and prostate cancer where adequate vitamin D support has been found to be beneficial.

## Abbreviations

SNP = single nucleotide polymorphism

D_3 _= cholecalciferol, vitamin D

25-OH-D_3 _= calcidiol

1,25-OH_2_-D_3 _= calcitriol

CYP24A1 = cytochrome P450, family 24, subfamily A, polypeptide 1

VDR = nuclear vitamin D receptor

IL12B = interleukin 12 B (cytotoxic lymphocyte maturation factor 2, p40)

RXRA = retinoid X receptor α

IL4R = interleukin 4 receptor

ADRB2 = ß2 adrenergic receptor

IL12RB1 = interleukin 12 receptor, ß1

IL10 = interleukin 10

GC = group-specific component (vitamin D binding protein)

CYP2R1 = cytochrome P450, family 2, subfamily R, polypeptide 1

CARD15 = caspase recruitment domain family, member 15 (NOD2)

SPP1 = secreted phosphoprotein 1, osteopontin (OPN, ETA-1, BNSP,...)

CYP27B1 = cytochrome P450, family 27, subfamily B, polypeptide 1

## Authors' contributions

M.W. initiated the study, applied for funding, developed protocols, trained investigators, planned laboratory analysis, did statistical analysis and wrote the report. J.A. did the clinical survey, C.B. did the SNP analysis, M.B. built serum and DNA bank and did the vitamin D assays together with E.A. who supervised also laboratory work and did functional assays. T.F-K. participated in the data analysis. All authors critically revised the paper.

## Conflicts of Interest

The author(s) declare that they have no competing interests.

**Figure 4:**
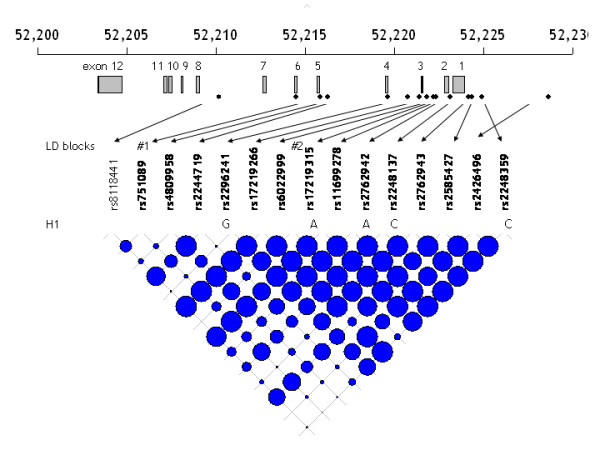
Genomic organization of CYP24A1 gene, location of genotyped SNPs, linkage disequilibrium between SNPs (with R2 indicated by bullet size) and LD block structure (highlighted by red boxes; rs2248359 was excluded from LD calculations for not being in HWE). SNPs indicated by ¶ were used to build haplotypes.
